# Downregulation of PAICS due to loss of chromosome 4q is associated with poor survival in stage III colorectal cancer

**DOI:** 10.1371/journal.pone.0247169

**Published:** 2021-02-17

**Authors:** Yusuke Kobayashi, Kensuke Kumamoto, Hirokazu Okayama, Takuro Matsumoto, Hiroshi Nakano, Katsuharu Saito, Yoshiko Matsumoto, Eisei Endo, Yasuyuki Kanke, Yohei Watanabe, Hisashi Onozawa, Shotaro Fujita, Wataru Sakamoto, Motonobu Saito, Tomoyuki Momma, Seiichi Takenoshita, Koji Kono

**Affiliations:** 1 Department of Gastrointestinal Tract Surgery, Fukushima Medical University School of Medicine, Fukushima, Japan; 2 Faculty of Medicine, Department of Gastroenterological Surgery, Kagawa University, Kagawa, Japan; 3 Department of Thyroid and Endocrinology, Fukushima Medical University School of Medicine, Fukushima, Japan; 4 Fukushima Medical University School of Medicine, Fukushima, Japan; Kawasaki Ika Daigaku, JAPAN

## Abstract

Phosphoribosylaminoimidazole carboxylase, phosphoribosylaminoimidazole succinocarboxamide synthetase (*PAICS*) encodes an enzyme that catalyzes *de novo* purine biosynthesis. Although *PAICS* has been implicated as a potential therapeutic target in several cancers, its clinical and prognostic significance in colorectal cancer (CRC) is not fully understood. To elucidate the roles of *PAICS* in CRC, we investigated *PAICS* expression in four cohorts consisting of a total of 1659 samples based on quantitative RT-PCR, microarray and RNA-seq analysis. Despite upregulated *PAICS* levels in tumor compared to those of normal mucosa, we found a decreasing trend of *PAICS* expression during tumor progression and metastasis. We conducted immunohistochemistry on 252 specimens, showing that PAICS protein was strongly expressed in the majority of CRCs, but not in adjacent mucosa. Notably, 29.0% of tumors lacked PAICS staining, and PAICS-negative expression in tumor had significant prognostic impact on poor cancer-specific survival in stage III CRC. Correspondingly, decreased levels of *PAICS* transcript were also correlated with poor relapse-free survival particularly in stage III patients, and this finding was robustly confirmed in three microarray datasets of a total of 802 stage II-III patients. Bioinformatics analysis of CRC tissues and cell lines consistently indicated a correlation between decreased *PAICS* expression and copy number loss of chromosome arm 4q. In conclusion, our results suggest that *PAICS* expression is downregulated during tumor progression due to genetic deletion of chromosome 4q in microsatellite stable but chromosomally unstable tumors. Furthermore, decreased expression of *PAICS* transcript or loss of PAICS protein may provide prognostic stratification for postoperative patients with stage III CRC.

## Introduction

Colorectal cancer (CRC) is one of the most common types of cancer, with an estimated annual incidence of over 1.8 million cases and over 880,000 deaths worldwide [1]. Curative surgery is the mainstay of treatment for the majority of patients with CRC, and then prognostic stratification and therapeutic decisions following surgery are highly dependent on pathological analysis of the resected specimen according to the TNM staging system [2]. Postoperative adjuvant chemotherapy is recommended for patients with stage III CRC, while it is only considered for a subset of stage II patients with high-risk characteristics [2–4]. However, a substantial proportion of these patients do not recur when treated with surgery alone, whereas others develop recurrence and death even after curative surgery followed by adjuvant chemotherapy [2,3]. Such clinically heterogeneous outcomes may reflect molecular heterogeneity among tumors. Colorectal carcinogenesis is driven by multiple genetic and epigenetic changes in tumor cells in association with genomic instability, namely, chromosomal instability (CIN) or microsatellite instability (MSI) [2,5,6]. Approximately 85% of CRCs develop through the accumulation of mutations, such as *APC*, *KRAS*, *TP53*, and *SMAD4*, often accompanied by CIN, displaying ongoing structural and numerical chromosomal changes [2,5–7]. Alternatively, 15% of CRCs with high-level microsatellite instability (MSI-H) exhibit hypermutation but typically lack CIN [5].

Unrestricted cell proliferation caused by defective cell cycle control is one of the most prominent features of cancer cells [8]. In rapidly dividing cells, such as cancer cells, *de novo* purine biosynthesis pathway is fundamental for cell proliferation in replenishing the purine pool, in which multiple enzymes are involved [9,10]. Phosphoribosylaminoimidazole carboxylase, phosphoribosylaminoimidazole succinocarboxamide synthetase (PAICS), an enzyme that catalyzes *de novo* purine biosynthesis, has recently been found to have roles in several solid cancers, including lung cancer [11], prostate cancer [12,13], bladder cancer [14], and pancreatic cancer [15]. These studies demonstrated that *PAICS* was upregulated in tumor tissues, dependent on a transcription factor, Myc, as compared to their normal counterparts [12,13,15]. Moreover, *PAICS* might be functionally involved in cellular proliferation, invasion and epithelial-mesenchymal transition, suggesting a therapeutic value of PAICS inhibiting strategies [11–15]. Notably, in patients with lung adenocarcinoma, high expression of *PAICS* in both transcript and protein levels was associated with poor prognosis in multiple cohorts based on microarray and immunohistochemistry [11]. The same group most recently reported that *PAICS* was increased in CRC tissues and was associated with proliferation, migration, invasion and metastasis using *in vitro* and *in vivo* models [16]. However, the clinical and prognostic values of *PAICS* expression in CRC is not well understood. Furthermore, genetic mechanisms underlying the dysregulation of *PAICS* remain largely unknown.

To address the possible role of *PAICS* expression in CRC, we utilized multiple independent cohorts based on qRT-PCR, microarray and RNA-seq, followed by immunohistochemistry for PAICS protein expression. The present study unexpectedly found a decreasing trend of *PAICS* expression along with tumor progression. We then demonstrated that decreased levels of *PAICS* transcript and loss of PAICS protein had significant impact on poor prognosis particularly in stage III CRC. Moreover, with the use of large-scale genomic and transcriptomic data for CRC tissues and cell lines, we revealed that decreased expression of *PAICS* was attributed to loss of chromosome arm 4q.

## Materials and methods

### Patient samples

This study enrolled 80 patients with stage I-IV primary CRC who underwent surgical resection at Fukushima Medical University (FMU) hospital between 2003 and 2011 without preoperative chemotherapy or radiotherapy. Both paired tumor and corresponding normal mucosa were collected from each patient, and the samples were immediately frozen after resection in liquid nitrogen and stored -80°C until RNA isolation (FMU-RNA cohort). We also enrolled another set of patients with CRC who underwent surgery at FMU hospital between 1990 and 2007 without preoperative therapy, and 252 stage 0-IV primary CRC patients with available formalin-fixed paraffin-embedded (FFPE) tumor sections were used for immunohistochemistry (FMU-FFPE cohort). Tumors were classified according to the Japanese Classification of Colorectal, Appendiceal, and Anal Carcinoma [17]. Clinical information was retrospectively obtained by review of medical records. The endpoint of interest was cancer-specific survival, which was defined as time from the date of surgery to the date of colorectal cancer-death. The study was conducted in accordance with the Declaration of Helsinki and was approved by the Institutional Review Board of Fukushima Medical University (Ref# 2117).

### Quantitative RT-PCR (qRT-PCR)

For the FMU-RNA cohort, total RNA was extracted from frozen specimens using TRIzol Reagent (ThermoFisher Scientific, Waltham, MA), and was reverse transcribed to cDNA using the SuperScript III First-Strand Synthesis System (ThermoFisher Scientific) according to the manufacturer’s instructions. qRT-PCR was carried out using Fast Start Universal Probe Master (Roche Diagnostics, Tokyo, Japan) on the 7500 real time PCR system in triplicate with TaqMan assays, including *PAICS* (Assay ID Hs00272390_m1), and *ACTB* (Hs99999903_m1) (ThermoFisher Scientific). Relative expression levels were determined with SDS software by the 2-ΔΔCt method as described by the manufacturer, with *ACTB* used as the calibrator gene.

### Microarray and TCGA data analysis

All microarray data are publicly available from the Gene Expression Omnibus (GEO) database (http://www.ncbi.nlm.nih.gov/geo). Normalized expression values were obtained from each dataset and were not processed further. If a gene is represented by multiple probe sets, the expression values of multiple probes were averaged. We obtained the following three microarray datasets based on different platforms, in which clinical and relapse-free survival information and MSI status, including MSI-H, MSI-L (low-level MSI) and MSS (microsatellite stable), were available. In the GSE41258 dataset, normal colon, adenoma, stage I-IV primary CRC and liver/lung metastasis samples were collected at Memorial Sloan-Kettering (MSK) Cancer Center and were analyzed on Affymetrix U133A (MSK cohort) [18]. The dataset GSE39582 is a large series of stage 0-IV CRC samples based on Affymetrix U133+2.0, collected for the Cartes d’Identité des Tumeurs (CIT) program (CIT cohort) [19]. We also utilized a stage II-III Norwegian CRC patient series who treated surgically at different hospitals in the Oslo region consisting of GSE24551 and GSE30378 using Affymetrix Human Exon 1.0 ST (Oslo cohort) [20]. To conduct Kaplan-Meir survival analysis in the three microarray cohorts (MSK, CIT and Oslo), we utilized the lowest quartile (the first quartile) as the cut-point to dichotomize patients in each stage into *PAICS*-Low or *PAICS*-High based on the expression of *PAICS* in each cohort. For TCGA data analysis, Illumina HiSeq RNA-seqV2 data for colorectal adenocarcinoma (TCGA, PanCancer Atlas) with clinical and genomic data (copy number alterations and loss of 4q) were downloaded through cBioPortal (http://www.cbioportal.org/) (TCGA cohort) [7,21,22]. We also used a human cell line dataset of large intestine from The Cancer Cell Line Encyclopedia (CCLE) obtained through cBioPortal (CCLE cohort) [21,23]. For the CCLE cohort, MSI status was obtained from Medico et al. [24]. Gene enrichment analysis was performed using The Database for Annotation, Visualization and Integrated Discovery (DAVID) Bioinformatics Resources6.8 (http://david.abcc.ncifcrf.gov/home.jsp), as described elsewhere [25,26]. DAVID Functional annotation tool was used for gene ontology (Biological Process), pathways (KEGG), and general annotations (Chromosome).

### Immunohistochemistry

Four-μm thick sections were deparaffinized and rehydrated, and endogenous peroxidases were blocked with 0.3% hydrogen peroxide in methanol. Sections were then incubated with Protein Block (Agilent Technologies, Santa Clara, CA). Rabbit polyclonal anti-PAICS antibody (ab151472, Abcam plc, Cambridge, UK) was incubated in a 1:400 dilution of 10 mM phosphate-buffered saline (PBS) containing Tween 20 (Sigma-Aldrich, St. Louis, MO) at 4°C overnight, and subsequently detected by a horseradish peroxidase (HRP)-coupled anti-rabbit polymer followed by incubation with diaminobenzidine (Dako EnVision+ System, Agilent Technologies). Sections were counterstained with hematoxylin. IHC slides were evaluated by two independent observers without knowledge of patients’ clinical information. Of 252 resected whole-tumor sections, 235 adjacent non-tumor tissues were also evaluated. PAICS immunoreactivity in tumor and in adjacent non-tumor mucosa was evaluated respectively, and it was considered PAICS-Positive when more than 5% of epithelial cells showed cytoplasmic staining of any intensity, while the rest were determined to be PAICS-Negative.

### Statistical analysis

Student’s t-test, Mann–Whitney *U* test, Fisher’s exact test, and Spearman’s correlation were used to determine differences in variables between two groups. Cumulative survival was estimated by the Kaplan–Meier method, and differences between two groups were analyzed by the log-rank test. Univariate and multivariate models were computed using Cox proportional hazards regression. All statistical analyses were two-sided and were conducted using Graphpad Prism v6.0 (Graphpad Software, Inc., La Jolla, CA) or SPSS Statistics version 26 (IBM Corporation, NY). *P*-values less than 0.05 were considered statistically significant.

## Results

### *PAICS* mRNA expression in tumor was decreased along with disease progression

**Fig 1:**
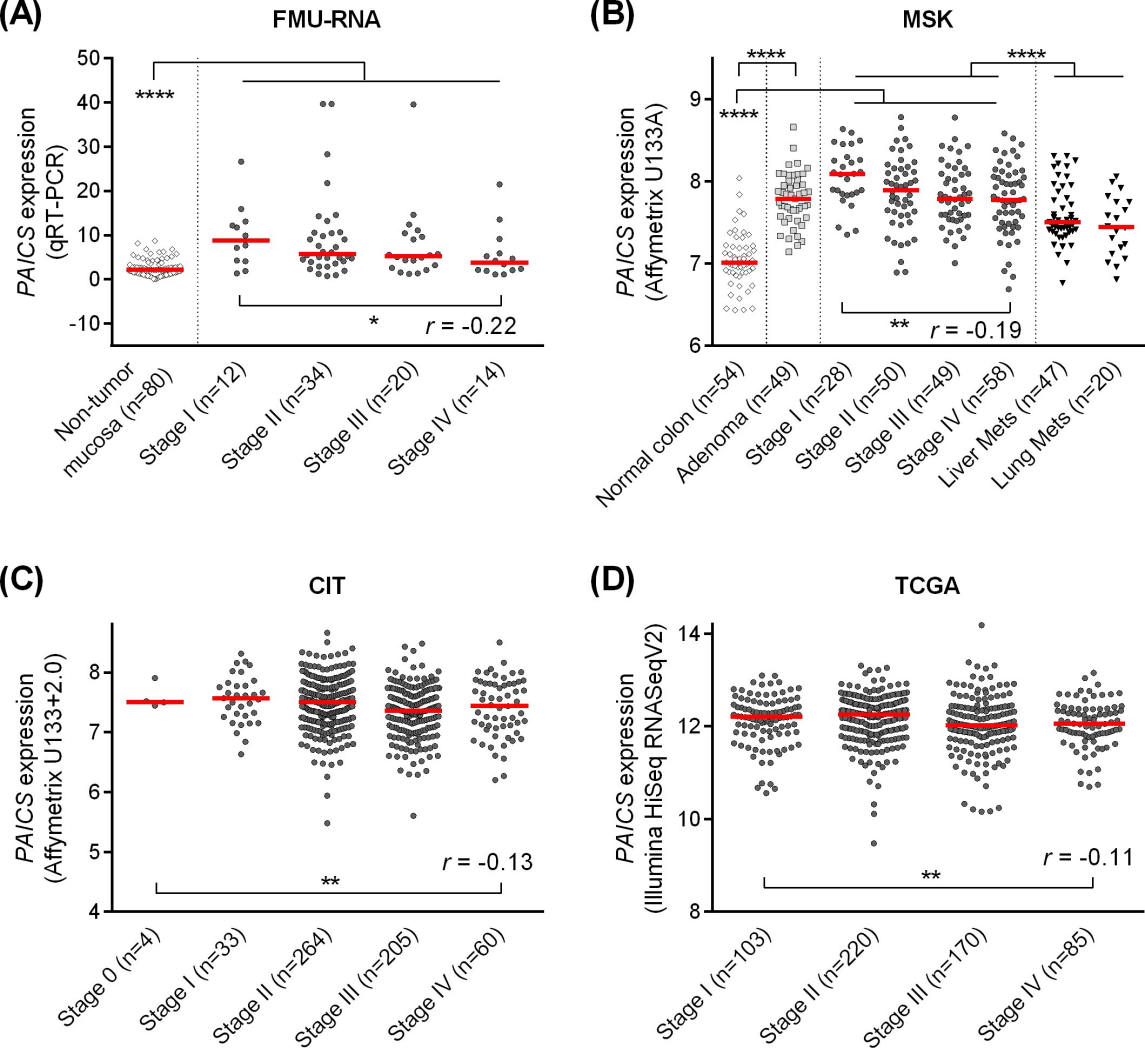
The expression of *PAICS* transcript in non-tumor mucosa, adenoma and colorectal carcinoma tissues. *PAICS* expression in the FMU-RNA cohort based on quantitative RT-PCR (A), the MSK cohort based on Affymetrix microarray U133A (B), the CIT cohort based on Affymetrix microarray U133+2.0 (C), and the TCGA cohort based on RNA-seq (D). *PAICS* was upregulated in cancer tissues compared to those of normal mucosa (A-B), but showed a decreasing trend along with tumor progression from stage I through stage IV primary tumors (A-D). *PAICS* levels were further decreased in metastatic tumors (lung or liver metastasis) than those of primary tumors (A). ****P<0.0001, **P<0.01, *P<0.05.

Multiple cohorts utilized in this study is summarized in S1 Table. We first investigated the expression of *PAICS* based on qRT-PCR using CRC and normal mucosa specimens obtained from 80 patients who underwent surgery at FMU hospital (FMU-RNA cohort, Fig 1). Consistent with the recent report by Agarwal et al. [16], we found that *PAICS* was significantly highly expressed in CRC tissues than those of normal mucosa (*P*<0.0001). However, when the correlation of *PAICS* expression with disease stage was analyzed, the expression of *PAICS* was found to be rather decreased with more advanced stages (r = -0.22, *P*<0.05, Fig 1A). In the MSK cohort analyzed on Affymetrix microarray, as shown in Fig 1B, we confirmed that *PAICS* was significantly overexpressed in colon adenomas and CRCs compared to those of normal mucosa (*P*<0.0001 and *P*<0.0001, respectively), and again we observed a significant decreasing trend of *PAICS* expression with progressive disease stages in primary tumors (r = -0.19, *P*<0.01). Correspondingly, the similar findings were obtained in both the CIT cohort (r = -0.13, *P*<0.01, Fig 1C) by Affymetrix microarray and the TCGA cohort (r = -0.11, *P*<0.01, Fig 1D) based on RNA-seq, showing a weak but significant negative correlation between the expression of *PAICS* and tumor progression. Also, metastatic tumors, including liver or lung metastasis, demonstrated a further decrease of *PAICS* expression compared to primary tumors in the MSK cohort (*P*<0.0001, Fig 1B). Of note, in most of the cohorts we analyzed, stage I tumors appeared to have the highest levels of *PAICS* among stage I-IV tumors.

### Loss of PAICS protein expression by immunohistochemistry was associated with poor survival in stage III CRC

**Fig 2:**
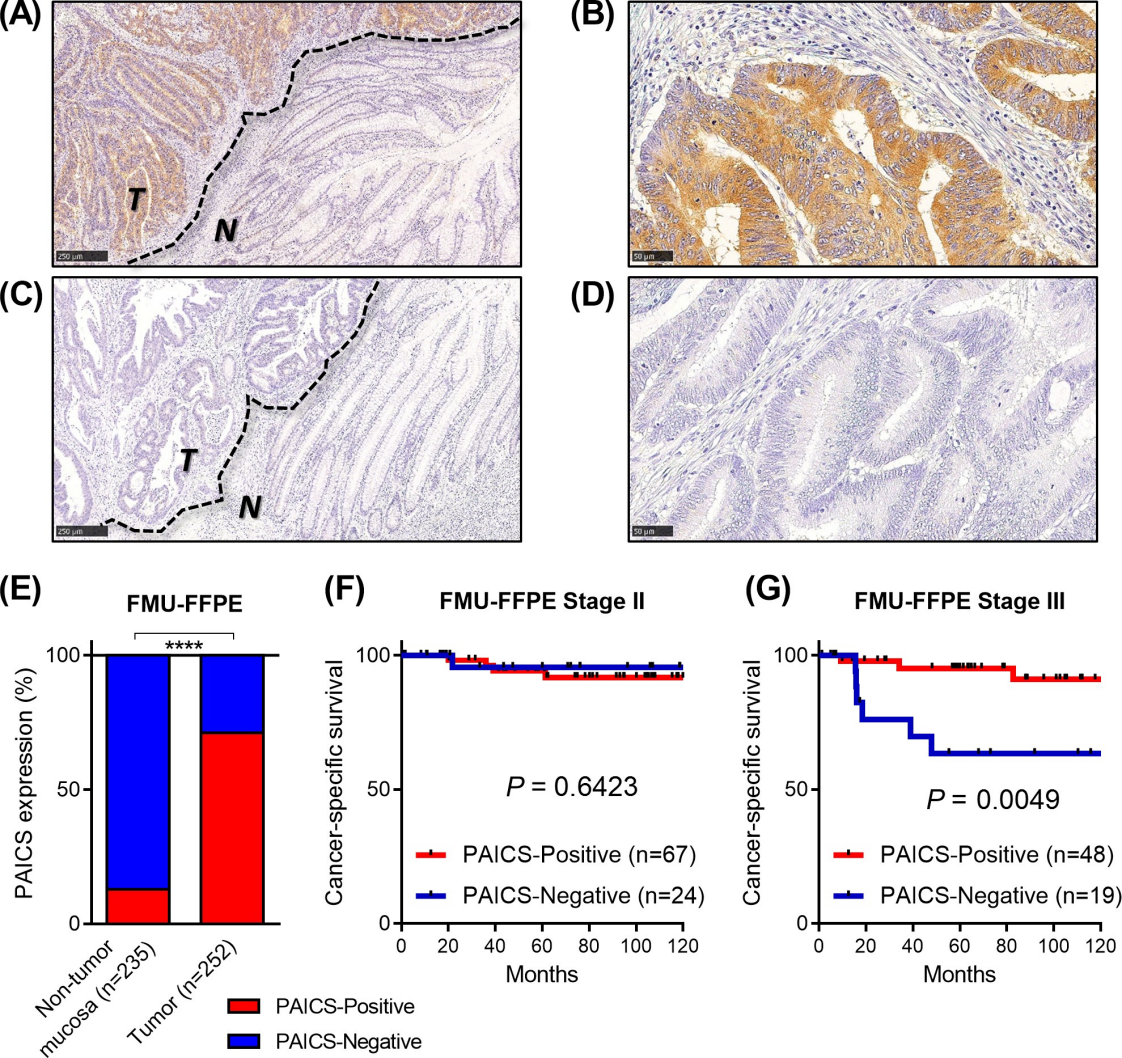
Immunohistochemistry for PAICS protein expression in resected colorectal cancer specimens in the FMU-FFPE cohort. (A-B) Representative images for PAICS-Positive tumors. PAICS staining was strongly demonstrated in the cytoplasm of tumor cells, but not observed in adjacent non-neoplastic epithelial cells or stromal cells. (C-D) Representative images for PAICS-Negative tumors, showing loss of PAICS staining in tumor cells. *T*; tumor, *N*; adjacent non-tumor mucosa. Scale Bars, 250μm (A and C) or 50μm (B and D). (E) PAICS was overexpressed in the majority of tumors compared to adjacent mucosa, whereas 31.0% of tumors were found to be PAICS-Negative expression. ****P<0.0001. (F-G) Kaplan-Meier curves for cancer-specific survival according to the expression of PAICS by immunohistochemistry in stage II (F) and stage III (G) patients.

We next examined the expression of PAICS protein in 252 resected CRC specimens by immunohistochemistry using the FMU-FFPE cohort (S1 Table and Fig 2). PAICS staining was rarely observed in adjacent non-neoplastic mucosal cells or stromal cells (Fig 2A–2D). In tumor tissues, PAICS expression was found in the cytoplasm of tumor cells (Fig 2A and 2B). Although moderate to strong cytoplasmic PAICS immunoreactivity was commonly seen in the majority of tumor tissues, we found that some tumors showed no PAICS staining, which appeared to be comparable to that of non-tumor mucosa (Fig 2C and 2D). We then classified specimens into PAICS-Positive (staining in more than 5% of epithelial cells with any intensity) or PAICS-Negative. Of 235 adjacent non-tumor tissues available for evaluation, only 30 (12.8%) were considered positive for PAICS expression, while 179 of 252 CRC tissues (71.0%) were found to be PAICS-positive (*P*<0.0001, Fig 2E), further confirming the overexpression of PAICS in tumor at protein levels. On the other hand, we found that 73 tumors lacked PAICS expression (PAICS-Negative, 29.0%). As demonstrated in Table 1, no significant association was found between the expression of PAICS protein and clinicopathological features, including age, gender, tumor location, tumor invasion, lymph node metastasis, distant metastasis or stage. By contrast, negative PAICS staining was more frequently observed in tumors with poorly-differentiated or mucinous histology, than those of well or moderately-differentiated (*P* = 0.011). We next sought to determine whether the expression of PAICS by immunohistochemistry was associated with survival in CRC. Kaplan-Meier analysis indicated that PAICS protein expression had no prognostic impact on cancer-specific survival in stage II patients (*P* = 0.6423, Fig 2F). However, in the analysis of stage III patients, negative-PAICS expression was significantly associated with worse cancer-specific survival (*P* = 0.0049, Fig 2G). We then conducted univariate and multivariate Cox analysis in stage II and III patients, showing that PAICS-Negative tumors had a nonsignificant trend toward poor prognosis, although not statistically independent (*P* = 0.079, S2 Table).

**Table 1:** Clinicopathological characteristics of colorectal cancer patients according to PAICS expression by immunohistochemistry.

		Total (n = 252)	PAICS expression		
			Negative	Positive	*P*
			n = 73 (29.0%)	n = 179 (71.0%)	
Age					0.861
	Mean±SD	65.9±11.9	65.8±12.0	66.1±11.6	
Gender					0.443
	Male	145	41 (28.3%)	104 (71.7%)	
	Female	107	32 (29.9%)	75 (70.1%)	
Location					0.476
	Right colon	85	26 (30.6%)	59 (69.4%)	
	Left colon	104	23 (22.1%)	81 (77.9%)	
	Rectum	63	24 (38.1%)	39 (61.9%)	
Histological differentiation					0.011
	Well-Moderately	224	59 (26.3%)	165 (73.7%)	
	Poorly-Mucinous	28	14 (50.0%)	14 (50.0%)	
Tumor invasion					0.059
	Tis	10	6 (60.0%)	4 (40.0%)	
	T1	25	9 (36.0%)	16 (64.0%)	
	T2	29	8 (27.6%)	21 (72.4%)	
	T3	106	31 (29.2%)	75 (70.8%)	
	T4	82	19 (23.2%)	63 (76.8%)	
Lymph node metastasis					0.356
	Absent	156	47 (30.1%)	109 (69.9%)	
	Present	96	26 (27.1%)	70 (72.9%)	
Distant metastasis					0.216
	Absent	212	64 (30.2%)	148 (69.8%)	
	Present	40	9 (22.5%)	31 (77.5%)	
Stage					0.149
	0	10	6 (60.0%)	4 (40.0%)	
	I	40	13 (32.5%)	27 (67.5%)	
	II	93	25 (26.9%)	68 (73.1%)	
	III	69	20 (29.0%)	49 (71.0%)	
	IV	40	9 (22.5%)	31 (77.5%)	

### Decreased *PAICS* mRNA expression identified poor prognostic patients with stage III CRC

**Fig 3:**
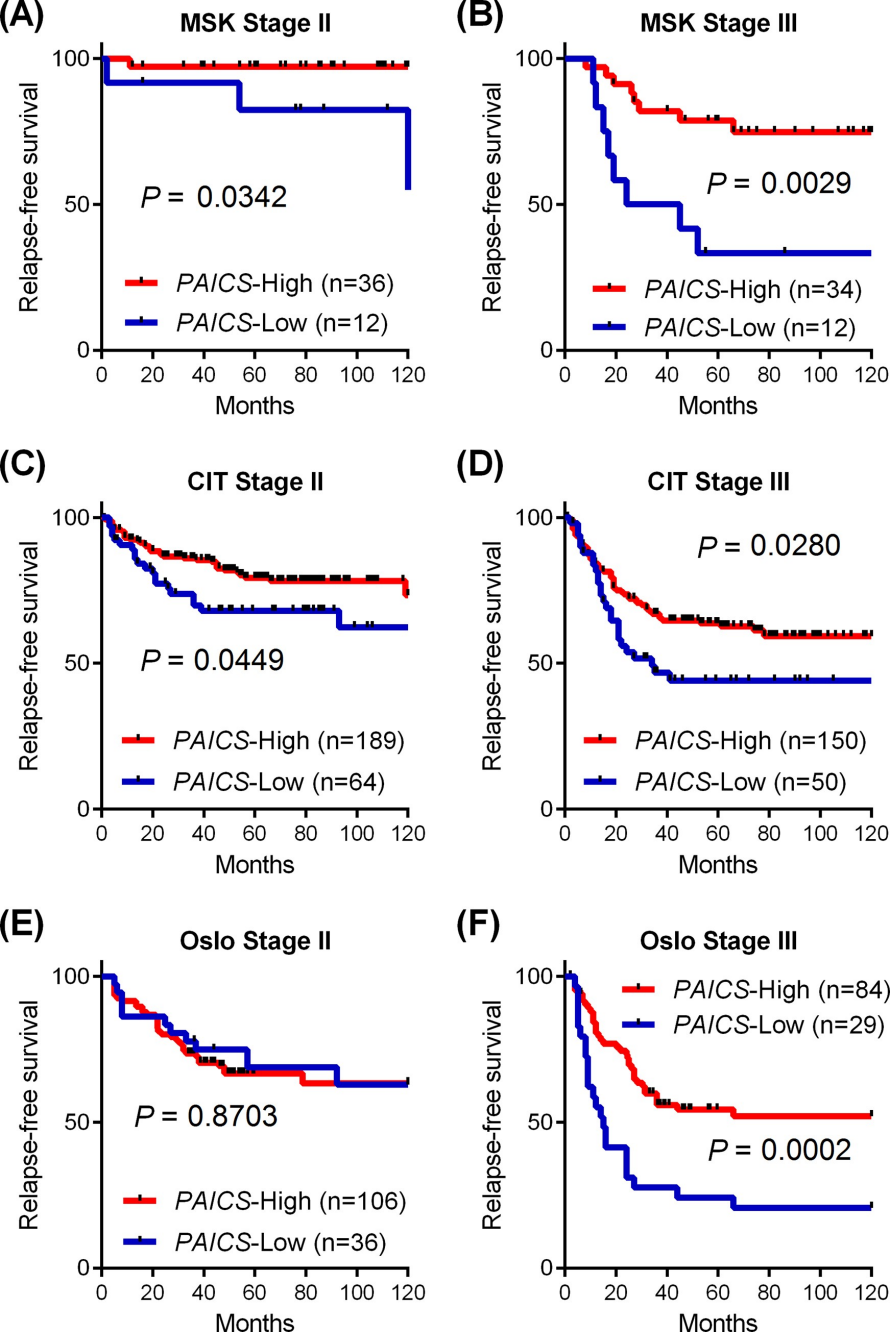
Decreased levels of *PAICS* expression was associated with poor survival in three independent cohorts. (A-F) Kaplan-Meier curves for relapse-free survival according to the expression of *PAICS* transcript in stage II (A, C and E) and stage III (B, D and F) patients in the MSK cohort (A-B), the CIT cohort (C-D) and the Oslo cohort (E-F). In each cohort, patients were dichotomized based on *PAICS* levels into *PAICS*-Low with the lowest quartile and *PAICS*-High with the higher 3 quartiles.

Since the lack of PAICS protein expression was associated with poor prognosis in the FMU cohort, we further addressed the prognostic role of *PAICS* mRNA expression in stage II and stage III patients using multiple independent datasets with survival information available, including the MSK (n = 94), the CIT (n = 453) and the Oslo cohorts (n = 255) (S1 Table). According to the expression levels of *PAICS*, tumors were dichotomized with the lowest quartile classified as *PAICS*-Low and the higher 3 quartiles classified as *PAICS*-High in each cohort for Kaplan-Meier analysis, as depicted in Fig 3A–3F. In patients with stage II CRC, decreased levels of *PAICS* expression were significantly or marginally associated with worse relapse-free survival in the MSK cohort (*P* = 0.0342, Fig 3A) and in the CIT cohort (*P* = 0.0449, Fig 3C), but it was not significant in the Oslo cohort (*P* = 0.8703, Fig 3E). Strikingly, prognostic impact of *PAICS* expression was particularly remarkable in patients with stage III CRC, demonstrating significant association between *PAICS*-Low tumors and poor relapse-free survival in the MSK cohort (*P* = 0.0029, Fig 3B), the CIT cohort (*P* = 0.0280, Fig 3D), and the Oslo cohort (*P* = 0.0002, Fig 3F).

### Decreased PAICS expression was associated with loss of chromosome arm 4q

**Fig 4:**
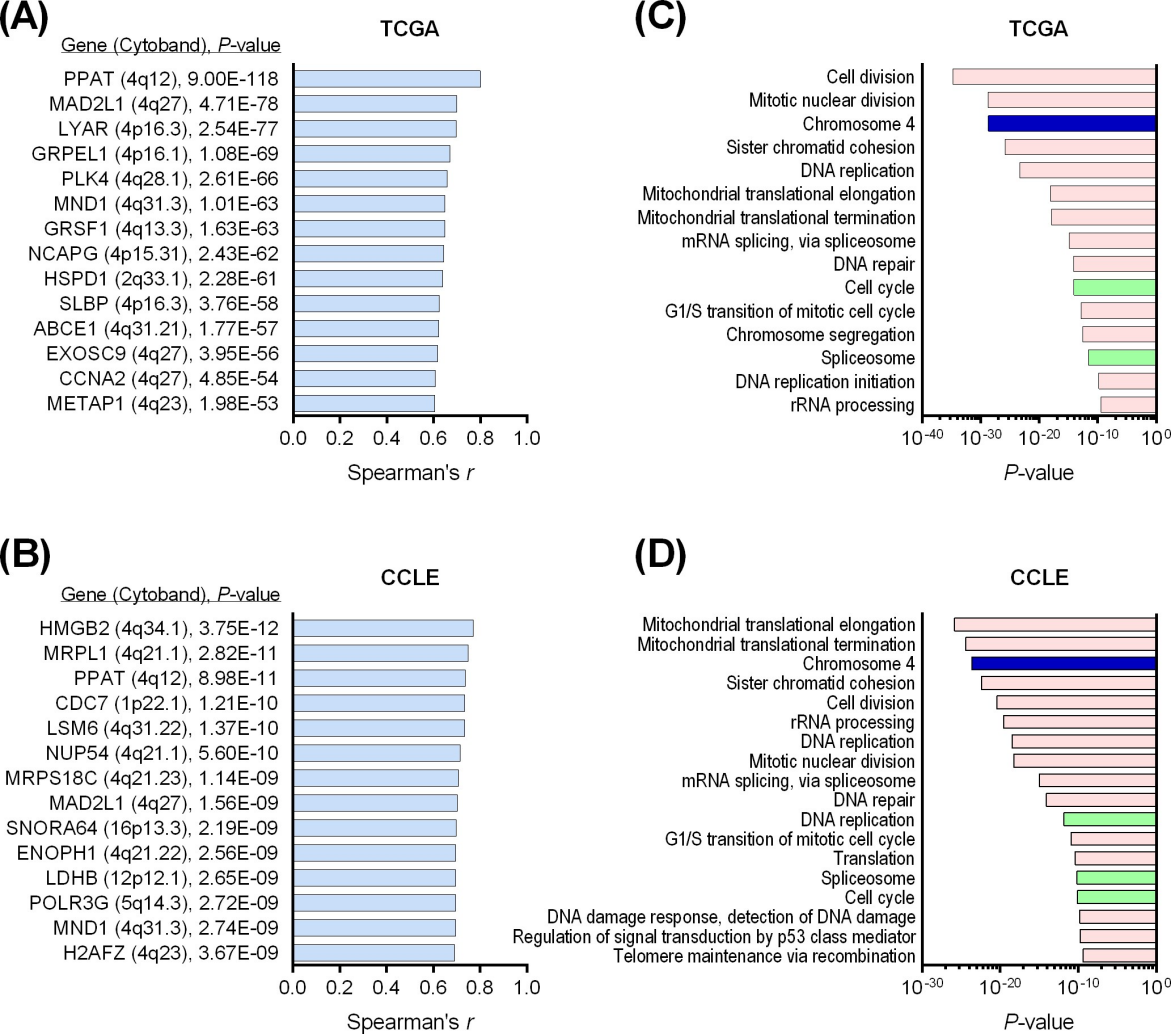
Co-expression analysis identified chromosome 4q genes were robustly correlated with *PAICS* expression in both colorectal cancer tissues and cell lines. (A-B) *PAICS*-co-expressed genes in the TCGA cohort (A) and the CCLE cohort of 57 colorectal cancer cell lines (B). The top ranked genes with Spearman correlation coefficient ≥0.6 and *P*<1.00E-8 are shown for each cohort. (C-D) Gene ontology analysis on 599 genes in the TCGA cohort and 843 genes in the CCLE cohort that were co-expressed with *PAICS* with Spearman correlation coefficient ≥0.4 identified significantly enriched GO terms (pink), pathways (green), and chromosomes (blue) in the TCGA cohort (C) and the CCLE cohort (D).

**Fig 5:**
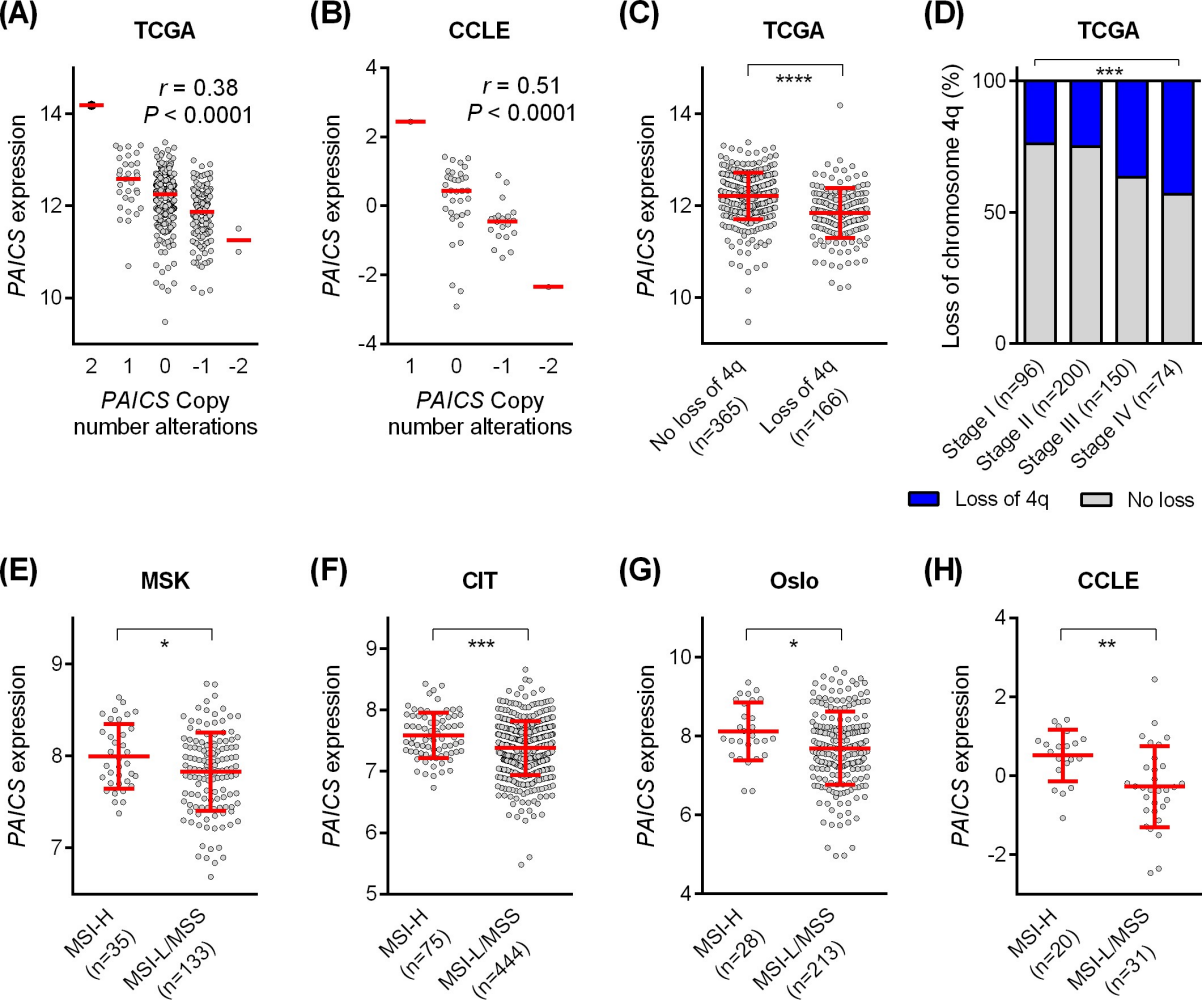
Decreased expression of PAICS correlated with loss of chromosome arm 4q. (A-B) Correlation between PAICS copy number and PAICS expression in the TCGA cohort (A) and the CCLE cohort (B). (C) Reduced levels of *PAICS* expression in tumors exhibiting loss of chromosome 4q in the TCGA cohort. (D) Increased occurrence of loss of chromosome 4q in advanced stage tumors in the TCGA cohort. (E-H) Decreased PAICS expression in MSI-L or MSS tumors compared to MSI-H tumors in the MSK cohort (E), the CIT cohort (F), the Oslo cohort (G) and the CCLE cohort (H). ****P<0.0001, ***P<0.001, **P<0.01, *P<0.05.

To elucidate the genetic mechanisms involved in the downregulation of *PAICS* in CRC, we utilized not only the TCGA cohort (n = 578) but also an additional microarray dataset of human CRC cell lines (the CCLE cohort, n = 57) (S1 Table), in which genomic data, including mutations and somatic copy number alterations (SCNAs) were available through cBioPortal. We conducted co-expression analysis to identify correlated genes the expression of which were significantly positively correlated with *PAICS* expression. Fig 4A and 4B demonstrated the correlated genes with Spearman correlation coefficient ≥0.6 and *P*<1.00E-8 in the TCGA cohort and the CCLE cohort, respectively. This revealed that several genes located on chromosome 4q were co-expressed with *PAICS* expression reproducibly in both cohorts, including *PPAT* (4q12), *MAD2L1* (4q27), and *MND1* (4q31.3). Gene ontology analysis using DAVID bioinformatics resources was performed on larger sets of *PAICS*-co-expressed genes with Spearman correlation coefficient ≥0.4 (599 genes in the TCGA cohort and 843 genes in the CCLE cohort). Significantly enriched GO (gene ontology) terms, pathways, and chromosomes are shown in Fig 4C for the TCGA cohort and in Fig 4D for the CCLE cohort (GO terms with *P*<1.00E-9 are shown). This analysis in both CRC tissues and cell lines consistently indicated that *PAICS* expression was regulated synchronously with a large number of genes located on chromosome 4. Also, *PAICS*-correlated genes were significantly enriched in tumor-related cellular processes, such as cell cycle, mitosis, cell division, DNA replication, DNA repair and RNA splicing. Moreover, we found that *PAICS* expression was significantly correlated with copy number alterations in both the TCGA cohort (Spearman r = 0.38, *P*<0.0001, Fig 5A) and the CCLE cohort (Spearman r = 0.51, *P*<0.0001, Fig 5B). On the other hand, there were few tumors harboring *PAICS* mutations of unknown significance. Those data clearly indicated that the dysregulated expression of *PAICS* was mainly attributed to arm-level or whole chromosome SCNAs. Correspondingly, chromosome arm-level analysis in the TCGA cohort demonstrated that decreased levels of *PAICS* expression was significantly associated with genetic loss of chromosome arm 4q (*P*<0.0001, Fig 5C). Moreover, the occurrence of loss of chromosome 4q was significantly more frequent in a later disease stage (*P*<0.001, Fig 5D), which is highly consistent with stage-dependent decrease of *PAICS* expression (Fig 1A–1D). As SCNAs correlate closely with CIN [7], we further investigated the association of MSI status with the expression of *PAICS*, showing that *PAICS* levels were significantly lower in MSI-L or MSS tumors that commonly exhibit CIN, compared with MSI-H tumors in the MSK cohort (*P*<0.05, Fig 5E), the CIT cohort (*P*<0.001, Fig 5F), the Oslo cohort (*P*<0.05, Fig 5G) and the CCLE cohort (*P*<0.01, Fig 5H).

## Discussion

In the present study, we addressed the clinical and prognostic significance of *PAICS* in CRC using multiple cohorts based on different platforms, leading to the identification of decreased *PAICS* expression and loss of PAICS protein as novel prognostic biomarkers for poor survival outcome, especially in patients with stage III CRC. Our findings also suggest that downregulation of *PAICS* can be attributed to copy number loss of chromosome arm 4q, principally accompanied by CIN.

We conducted qRT-PCR, microarray and RNA-seq analysis followed by immunohistochemistry and demonstrated that the expression of *PAICS* transcript and PAICS protein was significantly upregulated in the majority of tumor tissues, compared to that of non-tumor mucosa. Our finding is highly consistent with the previous report by Agarwal et al. showing that *PAICS* was overexpressed in CRC [16]. Likewise, the same group reported that *PAICS* expression was significantly higher in tumor tissues than that of their normal counterparts in several cancer types [11–15]. These studies suggested a tumor-promoting role of *PAICS* involved in proliferation, migration and invasion in cancer on the basis of *in vitro* and *in vivo* functional experiments of *PAICS* depletion, in which *PAICS* expression can be transcriptionally regulated by the oncogenic transcription factor, Myc [12,13,15,16]. Also, a metabolic profiling study of CRC revealed that *de novo* purine synthesis pathway genes, including *PAICS*, were highly upregulated even in adenomas and early stages of carcinogenesis, induced by Myc [27]. However, despite the significant upregulation of *PAICS* in adenoma and carcinoma, we unexpectedly found that stage I tumors showed the highest levels of *PAICS* and then it was gradually decreased with advancing disease stage in a stepwise manner. This negative correlation between *PAICS* levels and disease stage in primary tumors was confirmed in four independent cohorts of CRC using qRT-PCR, microarray and RNA-seq platforms. Moreover, metastatic tumors exhibited significantly lower *PAICS* expression than that of primary tumors. On the other hand, two independent analyses, including CRC tissues (TCGA) and CRC cell lines (CCLE), robustly indicated a significant correlation between *PAICS* DNA copy number and *PAICS* mRNA expression. Also, decreased *PAICS* expression was mostly due to arm-level or whole chromosome SCNAs, with a lesser contribution of focal SCNAs. Indeed, a large number of genes located on chromosome 4q showed strong co-expression patterns with *PAICS* in both the TCGA and CCLE cohorts, while *PAICS*-co-expressed genes were found to be highly enriched not only for genes involved in cell cycle, cell division, DNA repair and DNA replication but also for chromosome 4 genes. Consequently, *PAICS* expression declined in tumors that exhibited loss of chromosome 4q, demonstrating the coincidence of chromosome 4q loss and *PAICS* downregulation during tumor progression. Those data clearly suggest that loss of chromosome arm 4q is the responsible genetic mechanism for *PAICS* downregulation in CRC. We further speculate that loss of chromosome 4q can result from, at least in part, accumulated CIN, which is characterized by widespread imbalances in chromosome number and structure, commonly observed in microsatellite stable CRCs where genetic loss of chromosome 4q often occurs [7,28]. In fact, recurrent loss of 4q was scarcely found in hypermutated or MSI-H tumors but frequently correlated with non-hypermutated or non-MSI-H tumors that often exhibit CIN [7,29]. Correspondingly, in 4 independent cohorts we analyzed, decreased expression of *PAICS* was not common in MSI-H tumors, but was frequently observed in MSI-L/MSS tumors.

In the present study, using immunohistochemistry for PAICS, we identified a subgroup (29%) of tumors exhibiting loss of PAICS protein. Although these tumors were not significantly associated with common clinical features, such as tumor invasion, lymph node metastasis and distant metastasis, we found that stage III patients with PAICS-negative tumors had significantly worse prognosis than those of PAICS-positive. Furthermore, the similar prognostic effect was robustly reproduced at transcriptional levels, in which low levels of *PAICS* expression was significantly associated with poor prognosis in three independent cohorts of postoperative stage III CRC patients. Intriguingly, several studies investigated the prognostic effect of chromosomal aberrations in CRC, showing highly concordant results that loss of chromosome 4 or chromosome 4q was significantly associated with poor disease-free survival in CRC patients who underwent curative surgery [29–33]. Bardi et al. used tumor karyotype analysis, showing that loss of chromosome 4 was correlated with shorter disease-free survival [30]. Al-Mulla et al. demonstrated that loss of heterozygosity (LOH) on chromosome 4 was associated with metastatic recurrence [31]. Likewise, Brosens et al. reported that deletion of chromosome 4q, analyzed based on comparative genomic hybridization (CGH) arrays, had poor prognostic impact on disease-free survival [29]. Danner et al. also revealed that loss of chromosome 4q according to CGH analysis was associated with worse overall survival [33]. Given the close relationship between decreased *PAICS* expression and loss of chromosome 4q, their prognostic roles appeared to be highly consistent in demonstrating poor survival outcomes in postoperative patients with CRC. Therefore, decreased *PAICS* expression might be a surrogate for loss of chromosome 4q. Also, our results suggest that gene expression of *PAICS* and/or immunohistochemistry for PAICS protein could be used as prognostic tools for patients with postoperative stage III CRC that may help guide clinical decisions, including postoperative adjuvant chemotherapy and surveillance plans.

This study has some limitations. Particularly, functional implications of *PAICS* downregulation in late-stage CRCs remain to be determined. Concerning the potential tumor-promoting role of *PAICS*, transcriptionally induced by Myc in CRC [16,27], upregulated *PAICS* may confer growth advantage to epithelial cells and facilitate adenoma formation and early-stage colorectal carcinogenesis. By contrast, in the later stages of tumor progression, *PAICS* expression appeared to be diminished by genetic loss of chromosome 4q, correlating with poor prognosis. This suggests that concurrent loss of *PAICS* and other genes located at chromosome 4q may cooperatively contribute to CRC progression via dysregulation or impairment of key molecular mechanisms in cancer, for instance, cell cycle, cell division, DNA repair, and mitosis. Since arm-level deletions can cause co-suppression of multiple neighboring genes to synergistically contribute to tumor progression [34,35], functional roles of 4q loss might differ from those arising from *PAICS* downregulation. Therefore, future studies are necessary to confirm these results and to elucidate exact mechanisms by which chromosomal 4q aberrations affect tumor phenotypes and survival outcomes.

In conclusion, this study suggest that *PAICS* expression is downregulated during tumor progression mainly due to chromosome 4q loss particularly in microsatellite stable but chromosomally unstable tumors. Furthermore, the expression of *PAICS* transcript or PAICS protein may be clinically useful as prognostic biomarkers for stage III CRC after surgery.

## Supporting information

S1 Table Cohort characteristics. (PDF)

S2 Table Univariate and Multivariate Cox regression for cancer-specific survival in patients with stage II and III colorectal cancer. (PDF)
