# Supplementary material for: Downregulation of PAICS due to loss of chromosome 4q is associated with poor survival in stage III colorectal cancer
Source: PLoS One. 2021 Feb 17;16(2):e0247169. doi: 10.1371/journal.pone.0247169 (PMC7888640; doi:10.1371/journal.pone.0247169)
Supplement: S1 Table — (PDF) [file pone.0247169.s001.pdf]

Table S1. Cohort characteristics

Cohorts	total	FMU-RNA	FMU-FFPE	MSK	CIT	TCGA	Oslo	CCLE
Platforms		qRT-PCR	immunohisto chemistry	Affymetrix Human Genome U133A	Affymetrix Human Genome U133Plus2.0	Illumina HiSeq_RNASeqV2<br><br>Affymetrix SNP Array 6.0	Affymetrix Human Exon 1.0 ST	Affymetrix Human Genome U133Plus2.0<br><br>Affymetrix SNP Array 6.0
GEO accession		-	-	GSE41258	GSE39582	-	GSE24551<br>GSE30378	GSE36139
Total number of samples	2223	160	252	355	566	578	255	57
Normal colorectal mucosa	134	80	(235)	54	0	0	0	0
Colorectal adenoma	49	0	0	49	0	0	0	0
Stage 0 primary CRC	10	0	6	0	4	0	0	0
Stage I primary CRC	220	12	44	28	33	103	0	0
Stage II primary CRC	803	34	93	50	264	220	142	0
Stage III primary CRC	626	20	69	49	205	170	113	0
Stage IV primary CRC	257	14	40	58	60	85	0	0
Metastatic CRC	67	0	0	67	0	0	0	0
CRC cell line	57	0	0	0	0	0	0	57
MSI status								
MSI-H	220	0	0	35	75	62	28	20
MSS/MSI-L	1205	0	0	133	444	384	213	31
Unknown	548	80	252	17	47	132	14	6

Abbreviations: FMU, Fukushima Medical University; FFPE, formalin-fixed paraffin-embedded; MSK, Memorial Sloan-Kettering Cancer Center; CIT, Cartes d'Identité des Tumeurs program; TCGA, The Cancer Genome Atlas; CCLE, Cancer Cell Line Encyclopedia; GEO, Gene Expression Omnibus; qRT-PCR, quantitative RT-PCR; MSI, microsatellite instability; MSI-H, high-level microsatellite instability; MSS, microsatellite stable; MSI-L, low-level microsatellite instability
