# Supplementary material for: Downregulation of PAICS due to loss of chromosome 4q is associated with poor survival in stage III colorectal cancer
Source: PLoS One. 2021 Feb 17;16(2):e0247169. doi: 10.1371/journal.pone.0247169 (PMC7888640; doi:10.1371/journal.pone.0247169)
Supplement: S2 Table — (PDF) [file pone.0247169.s002.pdf]

Table S2. Univariate and Multivariate Cox regression for cancer-specific survival in patients with stage II and III colorectal cancer

Variable		Univariate			Multivariate†		
		HR	95% CI	*P*	HR	95% CI	*P*
PAICS (immunohistochemistry)	Negative vs Positive	2.67	0.94 – 7.62	0.066	2.84	0.89 – 9.13	0.079
Histological differentiation	Poorly-Mucinous vs Well-Moderately	5.48	1.90 – 15.81	0.002	3.33	1.00 – 11.11	0.050
Tumor invasion	T4 vs T1-T3	2.95	1.02 – 8.49	0.046	2.60	0.79 – 8.61	0.118
Lymph node metastasis	Present vs Absent	2.55	0.85 – 7.61	0.093	2.39	0.79 – 7.23	0.124
Age	Continous	1.02	0.97 – 1.07	0.427			
Gender	Female vs Male	0.50	0.17 – 1.43	0.196			
Location	Light colon/Rectum vs Right colon	4.52	0.59 – 34.57	0.146			
Adjuvant chemotherapy	Yes vs No	3.13	0.70 – 14.00	0.136			

† Variables with p-value less than 0.1 in the univariate analysis were evaluated in multivariate analysis
